# Targeting immune checkpoints on tumor-associated macrophages in tumor immunotherapy

**DOI:** 10.3389/fimmu.2023.1199631

**Published:** 2023-05-29

**Authors:** Shumin Xu, Chenyang Wang, Lingge Yang, Jiaji Wu, Mengshu Li, Peng Xiao, Zhiyong Xu, Yun Xu, Kai Wang

**Affiliations:** ^1^ Department of Respiratory and Critical Care Medicine, The Fourth Affiliated Hospital, Zhejiang University School of Medicine, Yiwu, China; ^2^ School of Medicine, Zhejiang University, Hangzhou, China

**Keywords:** immune checkpoints, tumor associated macrophages, PD-1, PD-L1, SIRP-α, CD39, CD73

## Abstract

Unprecedented breakthroughs have been made in cancer immunotherapy in recent years. Particularly immune checkpoint inhibitors have fostered hope for patients with cancer. However, immunotherapy still exhibits certain limitations, such as a low response rate, limited efficacy in certain populations, and adverse events in certain tumors. Therefore, exploring strategies that can improve clinical response rates in patients is crucial. Tumor-associated macrophages (TAMs) are the predominant immune cells that infiltrate the tumor microenvironment and express a variety of immune checkpoints that impact immune functions. Mounting evidence indicates that immune checkpoints in TAMs are closely associated with the prognosis of patients with tumors receiving immunotherapy. This review centers on the regulatory mechanisms governing immune checkpoint expression in macrophages and strategies aimed at improving immune checkpoint therapies. Our review provides insights into potential therapeutic targets to improve the efficacy of immune checkpoint blockade and key clues to developing novel tumor immunotherapies.

## 1 Introduction

Immunotherapy is one of the most effective approaches for the treatment of cancer. Immune checkpoint blockade (ICB) therapies, such as programmed cell death protein 1 (PD-1)/programmed cell death-ligand 1 (PD-L1) inhibitors and cytotoxic T-lymphocyte-associated protein 4 (CTLA-4) inhibitors, have exhibited promising therapeutic effects in the treatment of various malignancies, such as non-small cell lung cancer (NSCLC) and melanoma. Moreover, the field of immunotherapy has recently entered a new era with the emergence of novel immunotherapeutic strategies, such as chimeric antigen receptor T cells (CAR-Ts) and CAR macrophages (CAR-Ms) (1). However, challenges in immunotherapy based on immune checkpoint inhibitors (ICIs) still persist. Clinical trials have demonstrated that the response rates for immune drug monotherapy are low, ranging from 10% to 30% for most solid tumors, and that responses widely vary among tumors or individuals (2–4). Moreover, challenges in tumor immunotherapy, such as severe side effects and a lack of tumor-infiltrating lymphocytes, remain (5). Although adverse events associated with immunotherapy may indicate activation of the immune system, severe toxicity can be fatal (1). Therefore, developing strategies to improve clinical response rates and reduce drug toxicity is crucial.

Mounting evidence suggests that immunosuppression in the tumor microenvironment (TME) is a major challenge in maximizing the clinical efficacy of immunotherapy. The TME comprises a complex population of non-tumor cells that influence tumor immune evasion and affect the response rate and survival time to immunotherapy (6), including cancer-associated fibroblasts (CAFs) (7), endothelial cells (8), and immune cells, such as T cells and macrophages (9), etc. Macrophages exhibit high plasticity, whereby alterations in the environment can lead to alterations in the relative abundance of their two primary phenotypes, namely M1 and M2 macrophages. M1 macrophages, also known as classically activated macrophages, exhibit high expression of CD86, inducible nitric oxide synthase (iNOS), and other markers. Lipopolysaccharide and pro-inflammatory cytokines, such as interferon-γ (IFN-γ), can trigger M1 macrophages to secrete tumor necrosis factor-α (TNF-α), pro-inflammatory cytokines, including interleukin-1 (IL-1), IL-6, IL-8, and IL-12, and promote anti-tumor immune response. M2 macrophages, also known as alternatively activated macrophages, exhibit high expression of CD163, CD206, and other markers. Immunoregulatory cytokines, like IL-4, macrophage colony stimulating factor (M-CSF) can trigger M2 macrophages to secrete immunosuppressive factors, such as arginase 1 (Arg-1), IL-10, and transforming growth factor-β (TGF-β), and mediate the formation of immunosuppressive microenvironment (10–12).

Tumor associated macrophages (TAMs) recognized as one of the most crucial components of the TME, accounting for over 50% of the immune cells that infiltrate the TME (13, 14). The phenotypes of TAMs are extremely complex and diverse and M2 macrophages constitute the predominant TAM subpopulations. Moreover, certain TAM subpopulations may express both M1 and M2 signature molecules. Therefore, researchers mostly differentiate TAMs as M1-like and M2-like macrophages. TAMs have been implicated in a variety of processes in: (1) promotion of tumorigenesis and progression through cancer-associated inflammation (15), (2) introduction of tumor metastasis through involvement in epithelial-mesenchymal transition (EMT) regulation (16), angiogenesis and remodeling, and tumor cell intravasation (17–19), and (3) inhibition of T cell activity, secretion of cytokines such as IL-10, and expression of immune checkpoint ligands, thereby promoting immunosuppression of TME (20, 21). Additionally, TAMs regulate the anti-tumor immune response and are associated with low immunotherapeutic efficacy and decreased overall survival (OS) of patients with tumors (22, 23). Furthermore, the abundance of immune checkpoints in TAMs is closely related to the prognosis of immunotherapy in patients (24, 25). In addition, targeting immune checkpoints in TAMs enhances immunotherapeutic effects (26). These findings contribute to our understanding of immune checkpoints in TAMs and their role in immunotherapy, which is essential for developing effective immunotherapeutic strategies.

**Figure 1:**
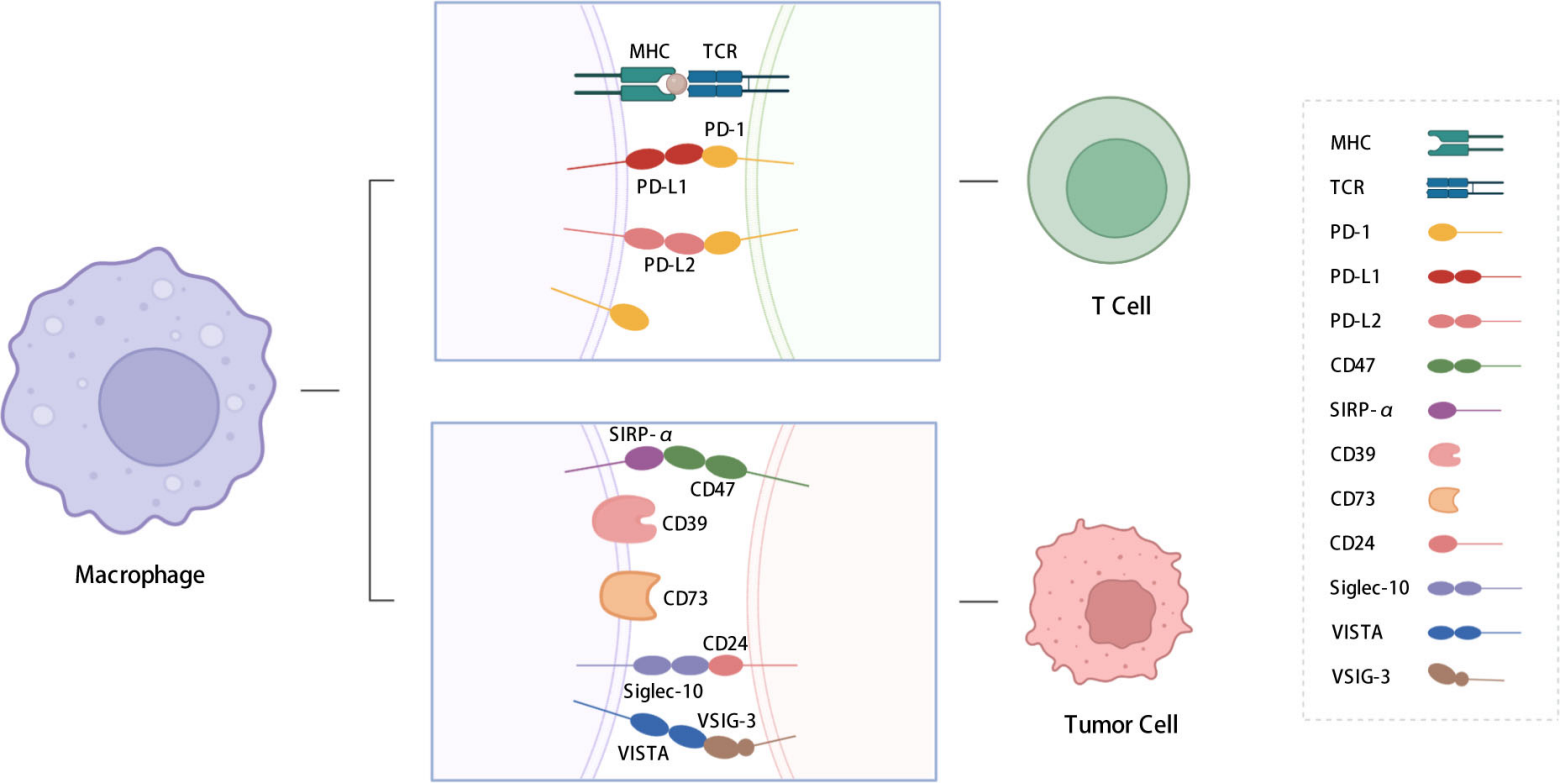
Immune checkpoints expressed on macrophages. We summarized the immune checkpoints expressed on macrophages including PD-1, PD-L1, PD-L2, SIRP-α, CD39, CD73, Siglec-10, VISTA, etc. PD-L1 and PD-L2 affect T cell functions; SIRP-α, CD39, CD73 and siglec-10 send “don’t eat me” signals to TAMs. PD-1: programmed cell death protein 1; PD-L1: programmed cell death-ligand 1; PD-L2: Programmed cell death-ligand 2; SIRP-α: signal regulatory protein α; VISTA: V-type immunoglobulin domain-containing suppressor of T cell activation; VSIG-3: V-set and Ig domain-containing 3; siglec-10: Sialic-acid-binding Ig-like lectin 10. The figure was created with BioRender.com.

In this review, we summarize the functions and regulatory pathways of immune checkpoint expression in TAMs. The immune checkpoint molecules discussed include PD-1, PD-L1, signal regulatory protein α (SIRP-α), CD39, CD73, V-type immunoglobulin domain-containing suppressor of T-cell activation (VISTA), and sialic-acid-binding Ig-like lectin 10 (siglec-10) (**Figure 1**). This review also presents an overview of strategies aimed at sensitizing immune checkpoint therapy through the application of drugs, inhibitors, and nanoparticles, as well as potential approaches to address the poor efficacy of immunotherapy, which are crucial for developing novel immunotherapeutic strategies.

## 2 PD-1/PD-L1 axis

PD-1, also known as CD279, is a member of the B7/CD28 family and is primarily expressed in activated CD4^+^ T cells, CD8^+^ T cells, B cells, NK cells, and other immune cells (27). PD-L1, also known as CD274 or B7-H1, is a ligand of PD-1 and is widely expressed in most tumor cells, macrophages, activated T cells, B cells, monocytes, and endothelial cells. The binding of PD-1 to PD-L1 recruits SH2 domain-containing protein-tyrosine phosphatase-2 (SHP-2) *via* the immunoreceptor tyrosine-based switch motif. This event subsequently triggers the dephosphorylation of spleen tyrosine kinase and PI3K, which then results in the inhibition of downstream AKT, ERK, and other signaling pathways; thereby suppressing the function of initial and effector T cells, inducing the production and activity of regulatory T cells, promoting tumor progression, and ultimately leading to a poor prognosis (28, 29).

The Food and Drug Administration (FDA) has approved numerous monoclonal antibodies (mAbs) against PD-1 and PD-L1 (nivolumab, pembrolizumab, atezolizumab, and durvalumab) for the treatment of various cancers, including melanoma and NSCLC (30–32). For example, pembrolizumab is used as the first-line treatment for lung adenocarcinoma (LUAD) or large-cell lung cancer with a PD-L1-positivity of ≥50%, and atezolizumab is used as the first-line treatment for patients with LUAD or sarcoma. Moreover, a combination of pembrolizumab and pemetrexed is used as a first-line treatment for patients exhibiting 1%–49% PD-L1 expression (33). ICI therapy has been reported to be beneficial for patients with PD-L1-positive NSCLC (30, 34). However, some patients with NSCLC strongly expressed PD-L1 and did not respond to PD-1/PD-L1 antibody therapy (35). In addition, patients with certain malignancies, such as breast, colorectal, renal cell, and prostate cancers exhibited inadequate response or low response rates to PD-1/PD-L1 antibody therapy (36–40). The reasons are related to various factors, such as the increasing expression of PD-L1, owing to various factors, such as the overexpression of PD-L1 (41), the lack of effective antigen presentation (42, 43), and the influence of TME, such as that of the Tregs, TAMs, and myeloid-derived suppressor cells (MDSCs). Furthermore, in clinical trials, hyper-progression has been observed, which indicated accelerated disease progression following ICB therapy, leading to poor prognosis (44, 45). The immunological mechanisms underlying hyper-progression are complex and may be primarily due to the destabilization of the immune editing and escape phases of tumors by ICIs, which ultimately promotes immune escape and results in accelerated tumor growth (46). ICB therapy is beneficial for patients with PD-L1-negative tumors, including NSCLC, kidney cancer, and other tumors, potentially owing to higher mutational and neoantigen load, possibly because of the heterogeneous expression of PD-L1 in the TME and distinct tumor histology (47, 48).

Thus, evaluation of tumor PD-L1 in isolation does not provide a comprehensive and accurate prediction of the effect of immunotherapy. Singhal et al. proposed that PD-L1 alone is an imperfect diagnostic biomarker (49). Therefore, combined analysis of various predictors, such as the addition of detecting macrophage PD-L1 levels, is needed to more faithfully reflect the composition of PD-L1 in the TME, predict the efficacy of PD-1/PD-L1 blockade, and maximize the therapeutic effects of immunotherapy in patients with cancer.

### 2.1 PD-1 on macrophages

PD-1 expression on TAMs varies across various types of tumors. In a study on gastric cancer, patients with low PD-1^+^ macrophage expression (PD-1^+^ macrophage < 0.85%) and high PD-1^+^ macrophage expression (PD-1^+^ macrophage ≥ 0.85%) exhibited five-year disease survival rates of 85.9% and 65.8%, respectively, suggesting that increased PD-1-positive macrophages in tumor tissues are associated with poor prognosis (50). Furthermore, PD-1 expression in TAMs was reported to be associated with immunosuppression and poor prognosis in colorectal cancer mouse models and primary human cancers (26, 51).

High expression of macrophage PD-1 modulates cell polarization and phagocytosis. In macrophages overexpressing PD-1, M1-related markers such as iNOS, were downregulated, whereas Arg-1, an M2-related marker, was upregulated. This may be attributed to the polarization of macrophages to the M2 phenotype by PD-L1-expressing T cells *via* the upregulation of the STAT6 pathway, which occurs as a result of the binding of macrophage PD-1 and reducing the release of cytokines, such as IL-1β, IL-12, from macrophages in the TME of pancreatic ductal adenocarcinoma mouse model (52). Similarly, the M2 population expressed obviously more PD-1 than the M1 population in colorectal cancer mice. PD-1 TAMs were foamy and present in a phagocytically inhibited state and the percentage of PD-1 TAMs was positively correlated with tumor size (26). In T cell lymphoma, the phagocytic inhibition caused by PD-1 overexpression in macrophages occurred because PD-1 signaling decreased the phosphorylation of STAT1/NF-κB in macrophages and inhibited M1 polarization (53). The NF-κB pathway has been found to regulate the expression of macrophage PD-1 in another research. Akito et al. reported significant circadian fluctuations in the expression of PD-1 on TAMs in melanoma mice, regulated by *Dec2*, which served as a molecular circadian clock. DEC2 inhibited the expression of macrophage PD-1 by suppressing p65 nuclear translocation, thereby affecting the anti-tumor effects of macrophages. By optimizing the time of administration, the anti-tumor efficacy of BMS-1, a PD-1/PD-L1 inhibitor, can be effectively enhanced. Particularly, BMS-1 could be administered at the time when PD-1 expression on TAMs is increased (54). The myeloid differentiation Factor 88 (MyD88)/IL-1R axis upregulated PD-1 expression on TAMs by promoting the phosphorylation of p65 and increasing its transcription of PD-L1. Consequently, the combination of MyD88 inhibitor and PD-1 mAbs resulted in a significant reduction in murine melanoma size and infiltration of F4/80^+^ CD11b^+^ macrophages, thereby increasing the recruitment of CD8^+^ T cells. Additionally, a reduction in PD-1 expression has also been observed, which effectively improved the prognosis (55).

### 2.2 PD-L1 on macrophages

#### 2.2.1 Effect of macrophage PD-L1 on tumor prognosis

Notably, compared to that on tumor cells, PD-L1 expression on TAMs is more closely associated with the efficacy of PD-L1/PD-1 blockade therapy. Recently, several studies have elucidated that, in hepatocellular carcinoma (HCC), ovarian small cell carcinoma, and breast cancer, the expression level of PD-L1 in TAMs is higher than that in tumor cells (56–58). In breast cancer, macrophage depletion has been shown to promote intertumoral infiltration of CD8^+^ T cells and reduced lung metastasis (58). Lin et al. found no difference in the effect of PD-L1 knockdown or overexpression on the efficacy of ICB therapy in colon, ovarian, melanoma, and lung cancer cell mouse models; however, they observed that functionally high expression of PD-L1 on macrophages was correlated with the efficacy of ICB and prognosis of patients (59). In patients with neuroblastoma and NSCLC, macrophage high PD-L1 expression was significantly associated with improved prognosis and OS (60). This phenomenon may be due to the presence of PD-L1^+^ macrophages, increased infiltration of CD8^+^ T cells, and higher expression of genes associated with active immune responses, such as *IFNG*, *GZMB* and *PRF1* (61, 62). However, patients with NSCLC with <4.7% PD-L1 expression on tumor cells or <6.3% PD-L1 expression on TAMs exhibited better five-year OS compared to patients with high PD-L1 expression in tumor cells or macrophages in a study by Sepesi et al. (63). Meanwhile, Xu-Monette et al. conducted an immunophenotypic analysis of 405 patients with *de novo* diffuse large B-cell lymphoma and showed that high PD-L1 expression on macrophages was associated with poor OS of patients with PD-1^hi^ CD8^+^ T cells, which is different from the previous study (64). In addition, another study showed that the ratio of PD-1 to PD-L1 on macrophages is correlated with patient prognosis. Patients with neuroblastoma with a PD-1/PD-L1 ratio >1 exhibited a lower survival rate; however, these patients benefited from PD-1 blockade therapy (65). Further investigation is required to determine if the specific reasons for these differences may be interrelated.

#### 2.2.2 Effect of macrophage PD-L1 on the immune microenvironment

Macrophage PD-L1 modulates the immunosuppressive nature of the TME through its function or by affecting T cells (66). Wagner et al. elucidated that PD-L1^+^ TAMs expressed both the pro-tumor markers CD204, CD206, and CD163, as well as the anti-tumor marker CD169, indicating that PD-L1^+^ TAMs have a dualistic nature (67). Zhu et al. demonstrated that macrophages with high PD-L1 expression were significantly associated with M2 polarization and secreted the typical chemokines, TGF-β and IL-10 (68). In glioblastoma, PD-L1 antibodies have been shown to enhance macrophage phagocytic activity by activating the ERK pathway (68) and to promote macrophage proliferation, survival, and activation, such as through the expression of the co-stimulatory molecules CD86 and MHC II (69). In clinical samples of ovarian cancer, PD-L1^+^ CD68^+^ macrophages have been elucidated to express higher levels of IL-10, IL-6, compared to PD-L1^-^ CD68^+^ macrophages; moreover, PD-L1 binding to T cells PD-1 has been demonstrated to induce apoptosis of T cells, thereby facilitating immune escape (70). Singhal et al. reported that PD-L1-expressing macrophages did not affect the interaction of effector T cells with tumor cells, and only act as “bystanders”; this may account for the lack of response to PD-1/PD-L1 blockade therapy in some patients with high PD-L1 expression (49). Macrophages expressing PD-L1 exhibit a dualistic nature, and their impact on the immune microenvironment is multifaceted, which primarily manifests as immunosuppressive functions. Following treatment with anti-PD-1/PD-L1 antibodies, macrophages depict enhanced phagocytic activity and activation, thereby reinstating their anti-tumor function, which is a crucial component of innate immunity.

#### 2.2.3 Regulation of PD-L1 expression on macrophages

**Figure 2:**
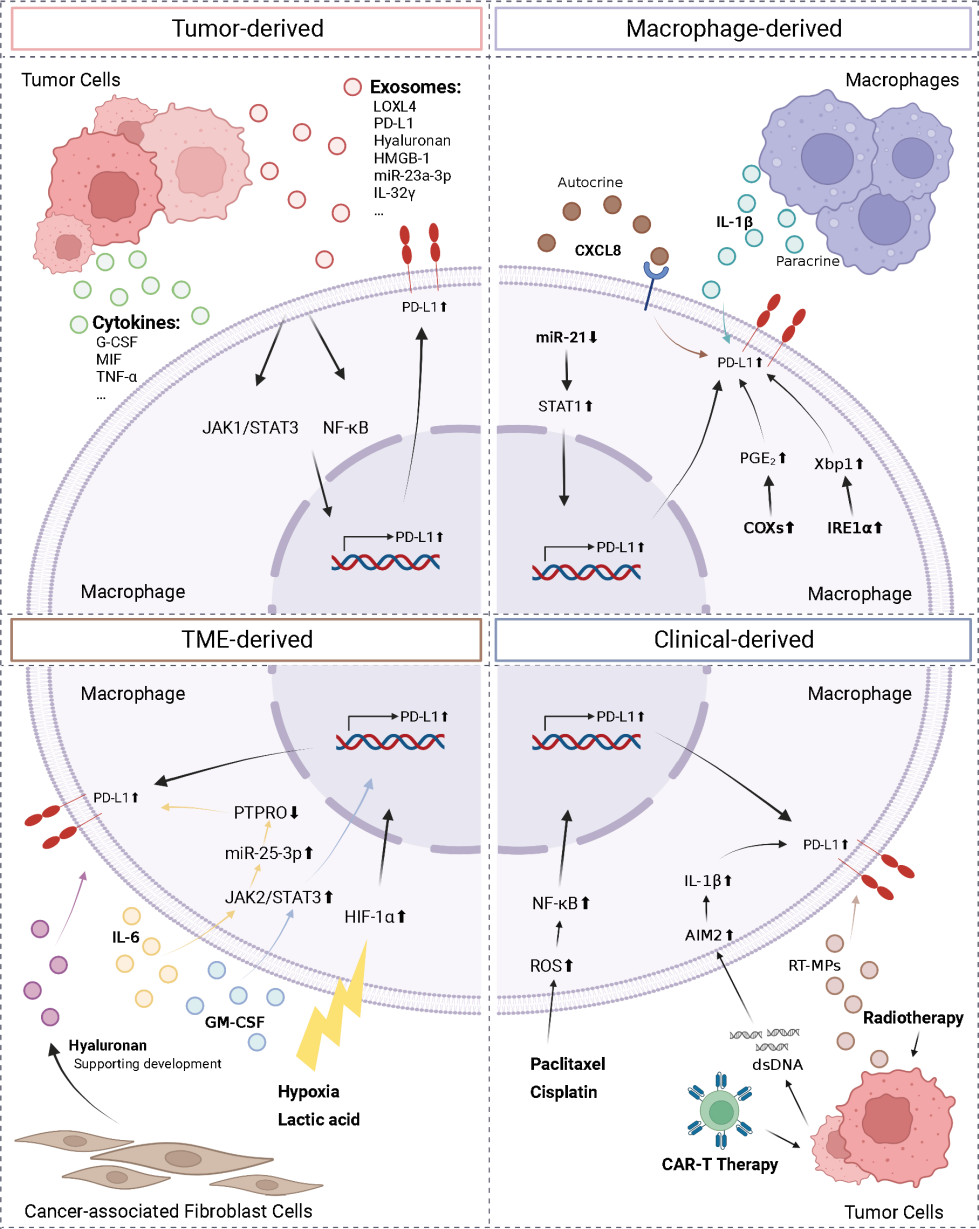
Regulation of macrophage PD-L1. We divided the factors affecting macrophage PD-L1 expression into four types: tumor-derived, macrophage-derived, TME-derived, and clinical-derived. G-CSF, granulocyte-colony stimulating factor; MIF, macrophage migration inhibitory factor; TNF-α, tumor necrosis factor-α; LOXL4, lysyl oxidase-like protein 4; HMGB-1, high-mobility group box-1; CXCL8, C-X-C motif chemokine ligand 8; PTPRO, protein tyrosine phosphatase receptor type O; AIM2, absent in melanoma 2; ROS, reactive oxygen species; IRE1α, inositol-requiring enzyme 1; PGE2, prostaglandin E2; COX2, cyclooxygenase-2. The figure was created with BioRender.com.

The complexity of the TME necessitates the identification of the factors regulating macrophage PD-L1 expression, which is required for establishing consistency and guidance in clinical treatment. Herein, we classified the factors affecting macrophage PD-L1 expression into four categories: tumor-derived, macrophage-derived, TME-derived, and clinical-derived (**Figure 2**).

Tumor cells can regulate PD-L1 expression on macrophages by secreting exosomes and cytokines. The conditioned medium of NSCLC cells and HCC cells co-cultured with macrophages has been reported to upregulate PD-L1 expression on macrophages (71, 72). HCC-derived exosomes secreted lysyl oxidase-like protein 4 (LOXL4) or PD-L1 or activated the STAT3 pathway, thereby promoting PD-L1 expression on macrophages and suppressing the function of CD8^+^ T cells (73–75). In addition, hyaluronan fragments in exosomes derived from HCC and lung cancer cells were thought to activate macrophage NF-κB signaling, thereby promoting PD-L1 expression (76, 77). Morrissey et al. reported that high-mobility group box-1 (HMGB-1) in exosomes derived from lung cancer cells activated the NF-κB pathway in macrophages *via* toll-like receptor 2 (TLR2); this activation promoted glycolysis, which led to lactate accumulation, and ultimately promoted upregulation of PD-L1 expression and induced an immunosuppressive phenotype in the macrophages (78). In addition, microRNAs in tumor secretory exosomes have been shown to regulate PD-L1 expression on macrophages. In HCC, endoplasmic reticulum stress resulted in the release of miR-23a-3p, which upregulated PD-L1 expression on macrophages and suppressed T cell function (79). Myeloma cells secreted IL-32γ-containing exosomes, which upregulated the expression of PD-L1 on macrophages by increasing the expression of 6-phosphofructo-2-kinase/fructose-2,6-bisphosphatase 3 (PFKFB3), ultimately facilitating immune evasion (80). Several factors secreted by tumor cells, such as granulocyte-colony stimulating factor (G-CSF), macrophage migration inhibitory factor (MIF), TNF-α, and autophagosomes (TRAPs), have been shown to upregulate PD-L1 expression on macrophages (58, 81–83).

In addition, factors secreted by macrophages have been shown to upregulate PD-L1 expression on macrophages. Knockdown of miR-21 in macrophages has been shown to upregulate PD-L1 expression. This occurred *via* the enhancement of IFN-γ-induced STAT1 signaling and the effect of PD-1 immunotherapy (84). Lin et al. found that macrophages can upregulate PD-L1 expression and suppress CD8^+^ T cell activity through autocrine C-X-C motif chemokine ligand 8 (CXCL8) (85). Macrophages produced active IL-1β through paracrine secretion following antibody-dependent cellular phagocytosis (ADCP), which was absent in melanoma 2 (AIM2) *via* FcγR signaling to promote macrophage PD-L1 expression, leading to immunosuppression (86). Additionally, the unfolded protein response in macrophages activated inositol-requiring enzyme 1 to promote IL-6 and PD-L1 expression (87). Factors that influence the TME included lactic acid accumulation and a hypoxic environment. Both factors activated the HIF-1α signaling pathway, upregulated PD-L1 expression on macrophages, and triggered the repolarization of macrophages to the M2 phenotype, thereby promoting immune escape (88, 89). In addition, in the TME, IL-6 upregulated miR-25-3p *via* the JAK2/STAT3/c-MYC signaling pathway to promote PD-L1 expression on macrophages, ultimately leading to increased immunosuppression (90). In breast cancer, granulocyte-macrophage colony stimulating factor (GM-CSF) derived from the inflammatory microenvironment enhanced PD-L1 expression on TAMs and inhibited T-cell activation by activating the STAT3 pathway (91). Dominguez et al. elucidated that epithelial tumor cells and CAFs also played roles in the upregulation of PD-L1 expression on macrophages (76).

Moreover, various clinical interventions and therapeutic measures affected PD-L1 expression on macrophages. In patients, PD-L1 expression was enriched on TAMs and upregulated following chemotherapy (92). Coincidentally, in a murine triple-negative breast cancer model, paclitaxel, an inhibitor of glutathione synthesis, induced the accumulation of reactive oxygen species, which then activated the NF-κB signaling pathway and promoted PD-L1 expression on macrophage (93). NSCLC cells treated by radiotherapy released microparticles (RT-MPs) that caused immunogenic death of tumor cells and upregulated PD-L1 expression on macrophages, polarized M2-like TAMs to M1-like TAMs, thereby enhancing the effect of subsequent combination therapy (94). Following treatment with CAR-T cells, tumor cells release dsDNA, which then activated AIM2 inflammatory vesicles, and induced upregulation of PD-L1 expression and 2,3-dioxygenase (IDO) on macrophages, thereby inhibiting the cytotoxicity of CAR-T cells and limiting the therapeutic effect of CAR-T. This effect can be reversed by anti-PD-L1 antibodies or IDO inhibitors (95).

Various interventions can be exploited to downregulate PD-L1 expression in macrophages. Exosomes secreted by HCC cells treated with melatonin have been shown to downregulate PD-L1 expression in macrophages by inhibiting STAT3 signaling and downregulating the secretion of IL-6, IL-1β, IL-10, and TNF-α by macrophages (73). In addition, JQ1, which is an inhibitor of the bromodomain and extra terminal domain, has been shown to downregulate PD-L1 expression in tumor cells, macrophages, and dendritic cells (DCs) and increase cytotoxic T-cell activity and IFN-γ secretion (96).

In summary, multiple factors, such as exosomes, cytokines, and metabolic substances, as well as certain clinical treatments, can affect PD-L1 expression on macrophages and induce an immunosuppressive microenvironment, thereby negatively impacting clinical outcomes. Alternatively, it can be argued that these factors or therapeutic interventions upregulate PD-L1 expression in macrophages, thereby rendering the TME to become more sensitive to ICB therapy. This approach can serve as a viable means of sensitizing tumors to ICB treatment. In conjunction with PD-1/PD-L1 antibody therapy, it can augment the infiltration and activity of CD8^+^ T cells and, thereby enhance anti-tumor immunity. Downregulation of PD-L1 expression could attenuate PD-1/PD-L1 axis-mediated immunosuppressive signaling, thereby generating a synergistic effect with PD-1/PD-L1 antibody therapy. This presents novel insights and potential avenues for the implementation of combination therapies.

### 2.3 PD-L2 on macrophages

TAMs have been found to exhibit low levels of PD-L2 expression compared to PD-L1 expression (97, 98); however, PD-L2 has been shown to exhibit a higher affinity for binding to PD-1 compared to PD-L1 (24, 99). Similar to PD-L1, PD-L2 has been reported to be expressed on macrophages and monocytes (97). In the HCC microenvironment, macrophages that express PD-L1 and PD-L2 are the predominant population of cells, with a positive correlation between PD-L1 and PD-L2 expression, as well as in cervical cancer TME (100, 101). However, no significant correlation between PD-L2 expression and OS/progression-free survival (PFS) outcomes has been found (101). These findings suggested that PD-L2 expression was often concomitant with PD-L1 expression and may be weaker compared to PD-L1 expression alone. Nonetheless, PD-L2 has been elucidated to play a role in tumor immune escape. A study on PD-L2-positive colon cancer by Tanegashima et al. revealed that anti-PD-L1 mAb treatment is effective, but not as efficacious as PD-1 mAb treatment, in both murine models and clinical settings (36). The combination of anti-PD-L1 and anti-PD-L2 mAbs could overcome this phenomenon. Additionally, Umezu et al. reported that PD-L1 mAbs upregulated PD-L2 expression in macrophages, and when mice were treated with anti-PD-L1 and PD-L2 inhibitors, their survival times were longer than each monotherapy, showing profound synergistic effects and long-term memory of the anti-tumor response.

The co-administration of anti-PD-L1 and PD-L2 mAbs addressed this phenomenon. Additionally, Umezu et al. reported that anti-PD-L1 antibodies upregulated PD-L2 expression on macrophages; moreover, they showed that mice with MC38 tumors, treated with anti-PD-L1 and PD-L2 inhibitors, exhibited prolonged survival rates compared to the use of either inhibitor alone, highlighting the profound synergistic effects of the combination therapy and long-term memory of the anti-tumor response (102).

### 2.4 Macrophage-targeting strategies involving the PD-1/PD-L1 axis

**Figure 3:**
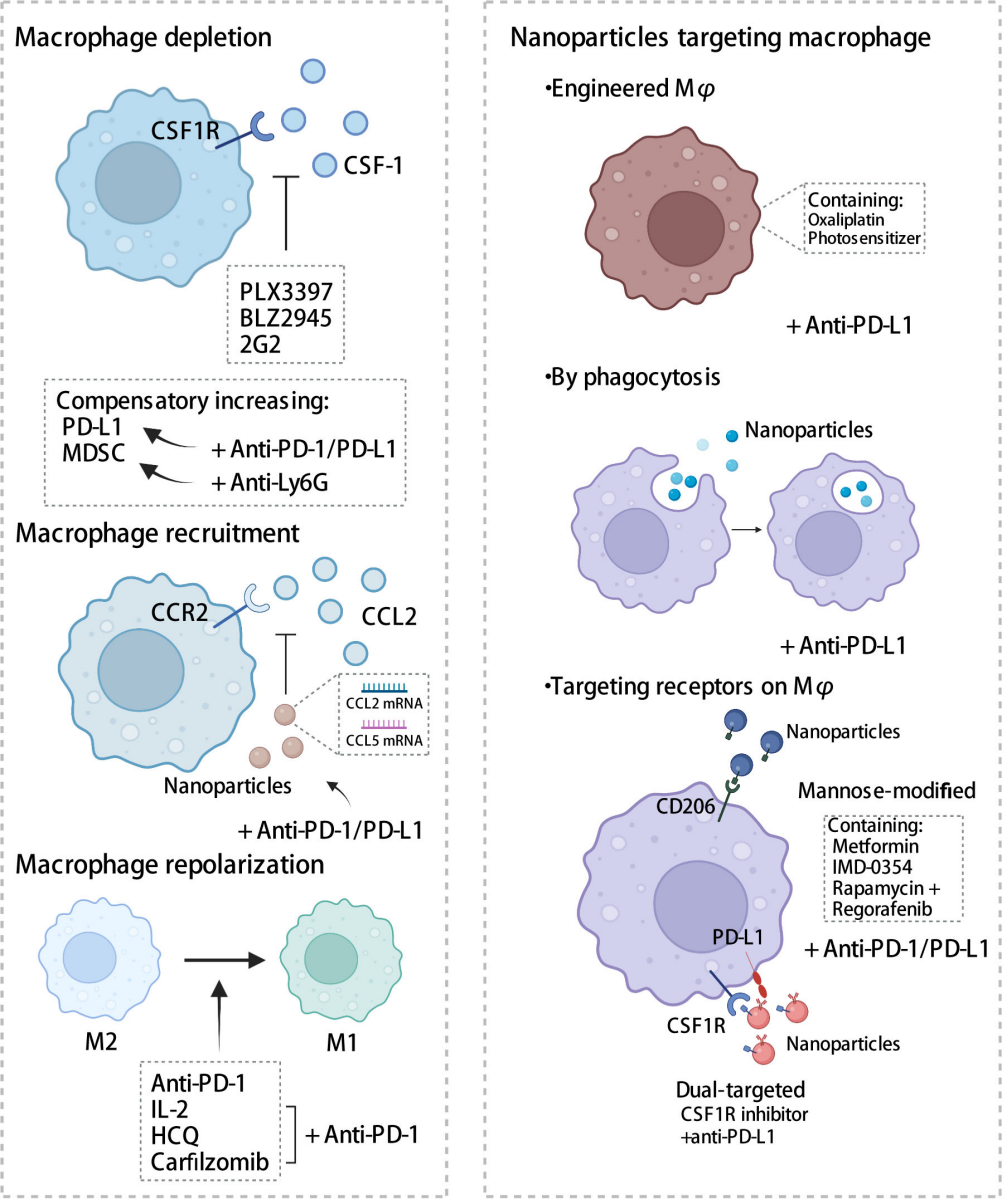
Strategies targeting macrophages combined with PD-1/PD-L1 antibodies. Therapies targeting macrophages include eliminating of TAMs, inhibiting recruitment, and reprogramming TAMs, in addition to the application of some nanomaterials. CSF-1, colony-stimulating factor 1; CSF1R, colony stimulating factor 1 receptor; MDSC, myeloid-derived suppressor cells; CCL2, C-C chemotactic factor ligand 2; CCR2, C-C chemotactic receptor 2; HCQ, hydroxychloroquine. The figure was created with BioRender.com.

Given the crucial role that TAM plays in the immune microenvironment, there is considerable interest in the implementation of TAM-targeted therapy in combination with ICI immunotherapy as a novel therapeutic approach (103). Herein, we summarize the most recent studies reporting the application of PD-1/PD-L1 blockade therapies in combination with TAM-targeted therapies (**Tables 1**, **2**). Macrophage-targeting approaches were classified as follows: (1) elimination of TAMs present in the TME; (2) inhibition of TAMs recruitment; (3) reprogramming of TAMs and (4) the application of certain nanoparticles (**Figure 3**).

**Table 1:** Clinical trials of PD-1 blockade combined with drugs targeting macrophages*.

Strategy	Cancer types	Clinical phase	Status/outcomes	Clinical identifier	Clinical Remark
Nivolumab + Cabiralizumab	PTCL	2	Active, not recruiting	NCT03927105	66.7% complete response rate at 4 months in 3 participants up to now
	Resectable BTC	2	Withdrawn	NCT03768531	No subject enrolled
Pembrolizumab + IMC-CS4	PAAD	1	Active, not recruiting	NCT03153410	No results posted
Pembrolizumab + ARRY-382	Advanced Solid Tumors	2	Terminated	NCT02880371	Halted due to insufficient efficacy
Pembrolizumab + PLX3397	Melanoma, NSCLC, HNSCC	1,2	Terminated	NCT02452424	Terminated early for insufficient evidence of clinical efficacy
PDR001 + BLZ945	Advanced Solid Tumors	1,2	Terminated	NCT02829723	No Results Posted
Nivolumab + BMS-813160	NSCLC, HCC	2	Recruiting	NCT04123379	No Results Posted
	Advanced PDAC	1,2	Recruiting	NCT03767582	Neoadjuvant and adjuvant trial
	PDAC	1,2	Recruiting	NCT03496662	Adverse events as anemia, nausea, fatigue, alopecia Increased objective response rate (ORR) when dose expansion
	PAAD	1,2	Active, not recruiting	NCT03184870	No Results Posted

* The data source from https://www.clinicaltrials.gov and the latest update date is March 9, 2023. Abbreviations, PTCL, Peripheral T Cell Lymphoma; BTC, Biliary Tract Cancer; NSCLC, Non-Small Cell Lung Cancer; PAAD, Pancreatic Cancer; HNSCC, Head and Neck Squamous Cell Carcinoma; HCC, Hepatocellular Carcinoma; PDAC, Pancreatic Ductal Adenocarcinoma; CRC, Colorectal Cancer; ICC, Intrahepatic Cholangiocarcinoma; HM, Hematologic Malignancies; MCC, Merkel-cell Carcinoma; SQC, Squamous Cell Carcinoma; MM, Multiple Myeloma; BRCA, Breast Carcinoma; HPV, Human Papillomavirus; HNC, Head and Neck Cancer; GAC, Gastric Adenocarcinoma; AML, Acute Myeloid Leukemia; MDS, Myelodysplastic Syndromes; NHL, Non-Hodgkin Lymphoma; MSS-mCRC, Microsatellite Stable Metastatic Colorectal Cancer; OCA, Ovarian Cancer; LMS, Leiomyosarcoma; CHN, Carcinoma of the Head and Neck; DLBCL, Diffuse Large B-Cell Lymphoma; FL, Follicular Lymphoma; MZL, Marginal Zone Lymphoma; MCL, Mantle Cell Lymphoma; CLL, Chronic Lymphocytic Lymphoma; BCL, B-Cell Lymphoma; EOC, Epithelial Ovarian Cancer; TNBC, Triple Negative Breast Cancer.

#### 2.4.1 Depletion of TAMs

The macrophage colony-stimulating factor signaling pathway, also known as the colony-stimulating factor 1 (CSF1) signaling pathway, plays a crucial role in macrophage differentiation and survival (51). The binding of CSF1 to its receptor, namely colony stimulating factor 1 receptor (CSF1R), promotes the survival and differentiation of human monocytes into macrophages, increased infiltration of TAMs, tumor invasion, metastasis, and angiogenesis (104). Blocking the CSF1-CSF1R axis has been shown to induce the repolarization of macrophages into M1 macrophages and enhance antigen presentation. In contrast, Yonemitsu et al. showed that CSF1 upregulates PD-L1 expression in macrophages by activating the STAT3 pathway (91). Therefore, CSF1R blockers in conjunction with antibodies against PD-1 or PD-L1 present a promising therapeutic strategy.

##### 2.4.1.1 PLX3397

In human and mouse lung cancer, PLX3397, a CSF1R inhibitor, reversed the long-term interaction between CD8 T cells and TAMs, thereby depleting TAM and inhibiting the migration and infiltration of T cells at the tumor site. In mouse models, PLX3397 treatment enhanced CD8^+^ T cell infiltration, thereby significantly improving the efficacy of PD-1 mAbs and delaying tumor progression (105). In HCC, osteopontin upregulated PD-L1 expression by activating the CSF1-CSF1R pathway in macrophages. Moreover, PLX3397 in conjunction with PD-L1 mAbs reduced TAM infiltration, increased intra-tumoral T cell activation, and prolonged the survival of HCC mice (106). PD-L1 in extracellular vesicles (TEVs) derived from colon and prostate cancer cells bound to PD-L1 mAbs and served as “decoys”, which were subsequently cleared by macrophages, causing the depletion of the antibodies and resulting in insufficient PD-L1 blockade and drug resistance (107). Upon employing PLX3397 to deplete macrophages, the non-phagocytized PD-L1 antibodies dissociated from TEVs favoring the binding between anti-PD-L1 mAbs and PD-L1 on tumor cells, thereby abolishing anti-PD-L1 therapeutic resistance. Preclinical studies mentioned above have shown a favorable synergistic effect of PLX3397 and PD-1/PD-L1 mAbs in suppressing tumors, as well as enhancing CD8^+^ T cell function. However, Shi et al. reported that the immunosuppressive state of the TME is ameliorated by PLX3397 through the reduction in the proportion of TAMs, rather than through the induction of their polarization; moreover, the combination of PLX3397 and PD-1 mAbs yielded undesirable anti-tumor efficacy and did not significantly improve the infiltration and activation of T cells; thus, this may be a limitation of this therapeutic approach (108). This suboptimal therapeutic outcome may be attributed to the dosage and the timing of the administration of the combination drugs. A clinical trial, NCT02452424, was designed to evaluate the safety and efficacy of PLX3397 (highest candidate dose, 800 mg/day) in combination with pembrolizumab; however, the trial was terminated prematurely owing to inadequate evidence of clinical efficacy. Adverse events associated with PLX3397 include alterations in hair color, fatigue, and elevated glutamate pyruvic, glutamic-pyruvic transaminase and blood alkaline phosphatase levels. PLX3397 has been shown to be generally well tolerated; however, notably, it exhibits hepatotoxicity, which may be due to the reduction of Kupffer cells (12, 109, 110).

##### 2.4.1.2 BLZ945

Studies have depicted that mesenchymal-like glioblastomas had abundant CD163^+^ TAMs, which dominated the immunosuppressive microenvironment owing to a high expression of PD-1/PD-L1 immune checkpoints and secretion of cytokines such as TGF-β, IL-10, and CSF1. To enhance the efficacy of PD-1 mAbs, researchers have combined PD-1 mAbs with BLZ945, a CSF1R inhibitor, in a humanized *ex vivo* model using patient-derived cells; this has resulted in the reduction in the number of CD163^+^ TAMs, enhancement of CD8^+^ T cell function, and induction of apoptosis of glioma cells (111). Magkouta et al. demonstrated that, in mesothelioma mouse models, BLZ945 inhibited the progression of mesothelioma, eliminated infiltration of TAMs and MDSCs, promoted the polarization of TAMs to the M1 phenotype, and stimulated the activation of CD8^+^ T cells; however, it also caused a compensatory increase in PD-L1 expression on macrophages and DCs and PD-1 immune escape signals on CD8^+^ T cells. In this instance, using PD-L1 antibodies reversed the limitations of the anti-CSF1R treatment, while exhibiting a synergistic effect (112). Preclinical studies above have suggested that BLZ945 could promote the polarization of M2 macrophages to the M1 phenotype. Currently, only two clinical trials pertaining to BLZ945 have been conducted. One of them, NCT02829723, is a phase I and II trial investigating the efficacy of BLZ945 as a monotherapy or in combination with PDR001, a PD-1 inhibitor, on advanced solid tumors. No study has reported any findings regarding its safety and efficacy.

##### 2.4.1.3 Others

Cui et al. validated that the combination of CSF1R antibody 2G2 and PD-1 antibody altered the immune profile in tissues; particularly, it increased T cell infiltration and the ratios of CD8/CD4 and prolonged survival of mice with glioma (113). However, Loeuillard et al. found that blocking TAMs with CSF1R mAbs resulted in compensatory accumulation of granulocyte-MDSCs (G-MDSCs) and mediated immune escape. In mice with cholangiocarcinoma, the efficacy of anti-PD-1 therapy is enhanced when Ly6G mAb was administered in combination with AFS98, a CSF1R mAbs, through the elimination of G-MDSCs and TAMs, respectively (114). **Tables 1**, **2** list several clinical trials investigating the efficacy of the combination of CSF1R inhibitors and PD-1/PD-L1 antibodies. Currently, these clinical trials aim to explore the safety and efficacy of the combination therapy and are in the recruitment phase. Confirmatory evidence from trials remains lacking.

**Table 2:** Clinical trials of PD-L1 blockade combined with drugs targeting macrophages*.

Strategy	Cancer types	Clinical phase	Status/outcomes	Clinical identifier	Clinical Remark
Durvalumab + SNDX-6352	Unresectable ICC	2	Active, not recruiting	NCT04301778	20% ORR in 8 months Adverse events as abdominal pain, fatigue, increased AST
	Solid or Metastatic Tumor, Advanced Malignant Neoplasm	1	Completed	NCT03238027	No Results Posted
Avelumab + DCC-3014	Sarcoma	1	Active, not recruiting	NCT04242238	No Results Posted To find the safest dose of DCC-3014
Durvalumab + LY3022855	Solid Tumor	1	Completed	NCT02718911	Well tolerated Limited clinical activity
Atezolizumab + Emactuzumab	Solid Cancers	1	Completed	NCT02323191	Manageable safety profile with increased fatigue and skin rash A considerable ORR (12.5%) in ICB-experienced NSCLC patients

* The data source from https://www.clinicaltrials.gov and the latest update date is March 9, 2023. The meaning of abbreviations in this table are the same as in **Table 1**.

In addition, a study reported that the elimination of macrophages may possibly result in a compensatory increase in resident glioma-associated microglia and promote tumor progression in glioblastoma (115). Therefore, further investigation is needed to develop optimal strategies for enhancing the anti-tumor effects of antibodies and reducing the impact of compensatory changes in immunosuppressive cells on their efficacy.

#### 2.4.2 Recruitment of TAMs

TAM recruitment is mediated by C-C chemotactic factor ligand 2 and its receptor type 2 axis (CCL2-CCR2 axis). Various cells, including tumor cells, endothelial cells, and fibroblasts (116), secrete CCL2, which promotes the recruitment of monocytes from the periphery towards the tumor site, leading to their differentiation into TAMs and subsequent tumorigenic development (117). In cervical cancer, mammalian target of rapamycin complex1 (mTORC1) upregulated CCL2 expression through protein phosphatase 2A (PP2A)-mediated dephosphorylation of forkhead box K1(FOXK1), thereby inducing M2 macrophage infiltration (118). In mouse models of esophageal cancer, macrophages recruited by the CCL2-CCR2 axis stimulated immunosuppressive pathways to induce immune evasion by increasing PD-L2 expression and triggering M2 polarization; this suggested the possibility of dual inhibition of the CCRL2-CCR2 axis and the PD-1/PD-L2 signaling pathway and thus, enhanced anti-tumor effects (119). Epithelial membrane protein 3 (EMP3) induced CCL2 secretion and expression of PD-L1 in glioblastoma multiforme cells and promoted the recruitment of TAMs of the M2 phenotype. The inhibition of TAM recruitment by knocking down EMP3 in combination with anti-PD-1 administration increased tumor apoptosis, improved the immunosuppressive state of the TME, and effectively prolonged the OS of mice (120). These findings of preclinical studies indicate some potential for developing a combination therapy involving CCR2 inhibitors and PD-1/PD-L1 blockades. Currently, four clinical trials designed for evaluating the safety and efficacy of nivolumab in combination with CCR2 inhibitor BMS-813160 are in the enrollment phase and the clinical efficacy and toxicity profile of this combination therapy remains unclear.

### 2.4.3 Reprogramming of TAMs

The plasticity of macrophages allows for the improvement of the immune infiltration in the TME through the repolarization of macrophages into M1-like anti-tumor macrophages using various therapeutic methods. This approach is a promising strategy for anti-tumor therapy.

Previous studies have reported that PD-1/PD-L1 axis in macrophages mediated M2 polarization and downregulated co-stimulatory molecules (121). In contrast, PD-1 mAb blocked PD-L1-induced macrophage M2 polarization and the expression of CD206 and TGF-β (122). Wang et al. revealed that, in melanoma mice, a combination treatment of IL-2 and PD-1 triggered transcriptional reprogramming of macrophages. In the treatment group, M1-like macrophage markers, such as *Il12a*, *Cd38*, and *Parp9*, were upregulated, whereas genetic features associated with M2-like macrophages, such as *Il10*, *Cd36*, *B4galnt1*, *Dgkz*, and *Gab1*, were downregulated; moreover, the combination treatment promoted tumor-specific T cell activation and tumor vascular normalization to induce tumor cell death (123). Hydroxychloroquine polarized macrophages from the M2- to the M1-like state, which was characterized by spindle-shape, increased pseudopods, and upregulated iNOS expression in melanoma mice; moreover, it induced macrophages to release IFN-β, thereby inducing T cell-mediated cytotoxicity and notably increasing the ratio of M1 macrophages following combination therapy with PD-1 antibody (124). Factors such as cytokines, metabolic status, exosomes, and drugs can affect the polarization of macrophages (125); however, the targets for macrophage reprogramming have not been well recognized; thus, further investigation of the alterations in the TAM polarization status changes in the TME is warranted.

As mentioned previously, regulating the number and function of macrophages can enhance innate immunity and exert anti-tumor effects (103), and blocking the interactions between PD-1 and PD-L1 could regulate the number, activity of T cells and improve adaptive immunity. This combination strategy may improve adaptive and innate immunity, thereby effectively impeding tumor progression.

#### 2.4.4 Nanoparticles

Owing to their highly phagocytic nature, nanoparticles are more readily engulfed by macrophages, wherein they exert their functions. Nanoparticles exert their functions within macrophages through various mechanisms: (1) by serving as drug delivery carriers through engineered macrophages, (2) through phagocytosis, and (3) by targeting receptors on macrophages.

##### 2.4.4.1 Drug delivery carriers *via* macrophages

Nanoparticles can be loaded with drugs and employed as vehicles for targeted delivery. Huang et al. developed a chemo-photodynamic therapy, wherein they designed engineered macrophages that were equipped with a photosensitizer and oxaliplatin to induce cell death of breast cancer cells. These engineered macrophages exhibited M1-type polarization, increased secretion of pro-inflammatory markers TNF-α and IL-6, and decreased the secretion of anti-inflammatory factor IL-10. In combination with PD-L1 antibodies, this approach resulted in enhanced anti-tumor immunity and improved OS in mice (126).

##### 2.4.4.2 Phagocytosis

Gadofullerene (Gd@C_82_) modified with β-alanine nanoparticles was rapidly internalized by TAMs and induced the repolarization of TAMs to the M1 phenotype by activating NF-κB, thereby increasing the infiltration of CD8^+^ T cells. In combination with PD-L1 blockade, this approach significantly shrunk breast cancer tumors in mice (127).

##### 2.4.4.3 Targeting receptors on macrophages

Wei et al. synthesized a nanoparticle (Man-MP) that is enriched with metformin (Met@Man-MP) and targets the mannose-modifying receptor on macrophages. Upon internalization by macrophages, the nanoparticle repolarized TAMs to the M1 phenotype. Moreover, Met@Man-MP improved the infiltration of T cells into the tumor through its unique ability to degrade collagen, which also facilitated the penetration of anti-PD-1 drugs. The combination of nanoparticles and anti-PD-1 drugs has been shown to significantly prolong OS in mice with HCC (128). Chen et al. designed a dual-targeted liposome delivery system that primarily targets both colon cancer cells and macrophages *via* anti-PD-L1 nanobodies and mannose ligands. The liposomes were loaded with rapamycin, a mTOR inhibitor, and regorafenib, an anti-angiogenic drug. The system induced M2 macrophages to transition to the M1 phenotype by inhibiting the phosphorylation of STAT3, thereby inhibiting macrophage-induced angiogenesis, promoting CD8 T cell infiltration into the TME, and significantly inhibiting the growth of colon cancer in mice (129). By incorporating a CSF1R inhibitor and PD-L1 antibody on the surface of nanoparticles, Ramesh et al. designed a lipid nanoparticle, namely α-PDL1-CSF-LNP, that targets PD-L1-expressing TAMs. The nanoparticles upregulated phagocytosis, repolarized macrophages to the M1 phenotype, and recruited CD8^+^ T cells, with a significant increase in IFN-γ levels. In a mouse melanoma model, α-PDL1-CSF-LNP demonstrated significant anti-tumor efficacy (130). Nanoparticles are mainly used in preclinical studies to deliver drugs by various methods for better anti-tumor effects, showing promising prospects and further researches are needed to determine their efficacy, safety and tolerability in complex TME in human.

Collectively, we reviewed the expression, function, and regulatory pathways of the PD-1–PD-L1 axis in macrophages and summarized strategies aimed at improving immune checkpoint therapy through the application of drugs, inhibitors, and nanoparticles that target macrophages. The combination therapeutic strategies targeting PD-1/PD-L1 on TAMs synergistically improve the number and function of immune cells in the TME, thereby evoking anti-tumor immunity against solid tumors and facilitating the transition from “cold tumor” to “hot tumor.”

## 3 CD47/SIRP-α axis

CD47, also known as integrin-associated protein, is widely expressed in normal cells. CD47 ligands include signal regulatory protein α (SIRP-α), thrombospondin-1 (TSP-1), and integrins (131). SIRP-α is a member of the SIRP family, which constitutes of transmembrane glycoproteins, and its binding to CD47 is crucial for macrophage phagocytosis regulation. Upon binding of CD47 to SIRP-α in macrophages, two typical immunoreceptor tyrosine-based inhibitory motifs (ITIMs) at the tail end of SIRP-α are phosphorylated. Phosphorylated ITIM recruited and activated tyrosine phosphatases, particularly SHP⁃1 and SHP⁃2, which inhibited myosin accumulation in phagocyte synapses and limited the phagocytic activity of macrophages, thereby serving as a “don’t eat me” anti-phagocytic signal (132, 133). This combination also inhibited macrophage-mediated programmed cell removal and elimination of red blood cells that were mediated by complement receptors; this served to prevent the immune system from clearing normal cells (131, 134). Moreover, the binding of CD47 to SIRP-α inhibited integrin activation, thereby limiting macrophage proliferation in the vicinity of phagocytic targets and ultimately inhibiting phagocytosis (135). In addition, the regulation of macrophage phagocytosis is governed by the ratio of the activating ligand FcR to the inhibitory ligand SIRP-α. Fc receptor (FcγR) activation is inhibited upon binding of CD47 to SIRP-α, facilitated by dephosphorylated immunoreceptor tyrosine-based activation motifs (ITAMs) located adjacent after recruiting SHP-1 (136). Additionally, CD47 was expressed within macrophages, where it bound to SIRP-α expressed on the same macrophage. Inhibition of this cis interaction can induce hyper phagocytosis of macrophages (137).

**Figure 4:**
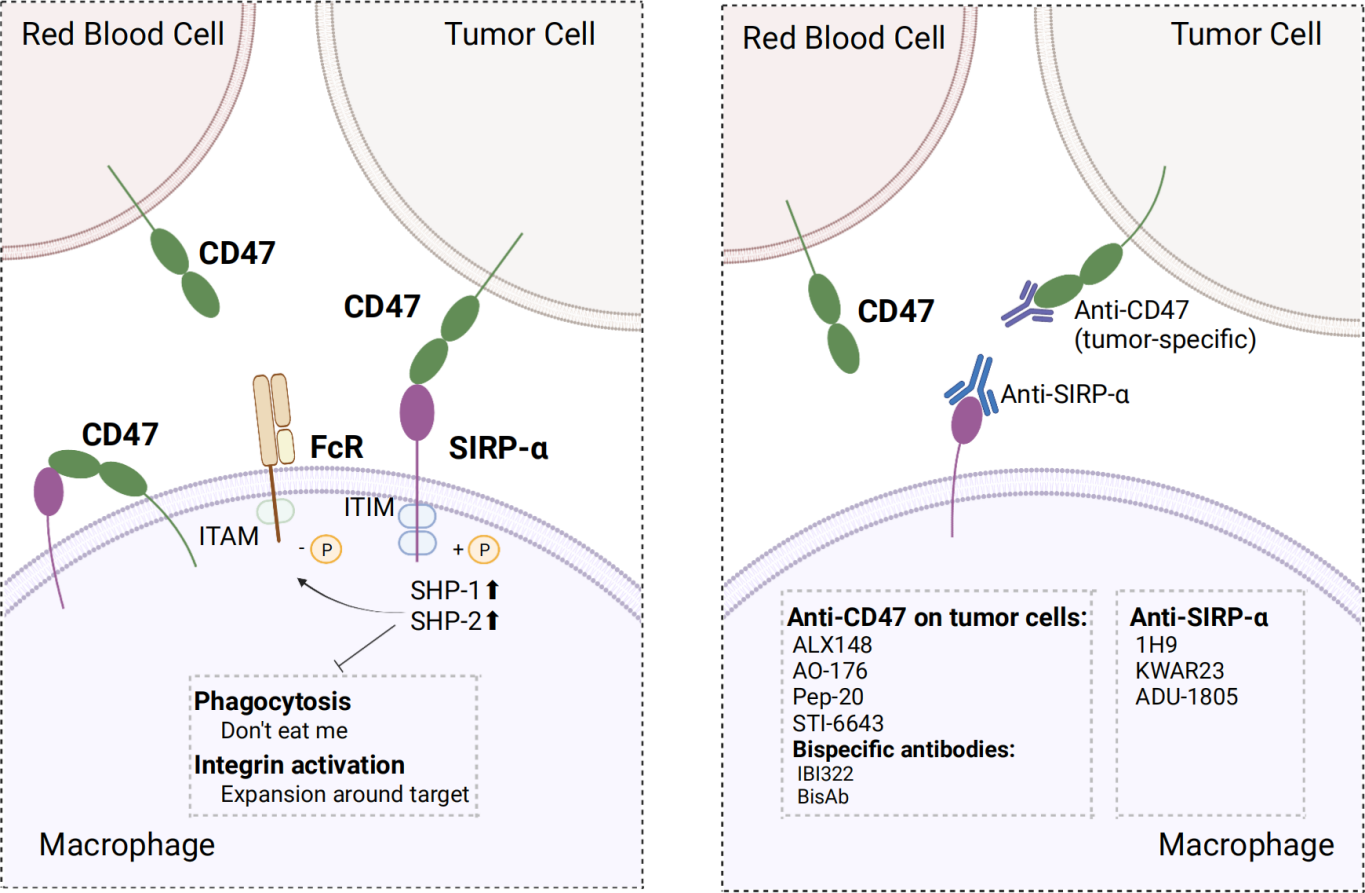
Mechanism of action of CD47 and SIRP-α and optimal strategy. Left, CD47 binds to SIRP-α in macrophages and provide “don’t eat me” signals by different mechanisms; Right, screening for antibodies that do not bind erythrocytes, using SIRP-α inhibitors or antibodies, and developing bispecific antibodies make strategies to optimize target treatment. ITIM, immunoreceptor tyrosine-based inhibitory motif; ITAM, immunoreceptor tyrosine-based activation motifs; SHP-1/2, SH2-containing tyrosine phosphatase 1/2. The figure was created with BioRender.com.

CD47/SIRP-α axis is a well-established innate immune checkpoint. CD47 is often overexpressed on tumor cells and binds to SIRP-α expressed on macrophages to deliver the “don’t eat me” signal to macrophages, which ultimately results in the evasion of immune surveillance; this phenomenon has been correlated with poor prognosis (138–140). Direct blockade of CD47 on tumor cells *via* the binding of the Fc segment of CD47 IgG1 antibody to FcγR on macrophages neutralized the inhibitory CD47 signaling and activated antibody-dependent cell-mediated cytotoxicity (ADCC) and complement-dependent cytotoxicity (CDC), which has a significant inhibitory effect on tumor cells, such as leukemic cells in non-human primate (141). Several clinical trials and preclinical studies investigating drugs that promote phagocytosis by blocking the CD47 axis are currently ongoing or have been concluded. Relevant clinical trials are summarized in **Table 3**. Nevertheless, using CD47 antibodies to treat tumors presents a challenge. Given the widespread expression of CD47, certain anti-CD47 antibodies exhibited similar targeting of normal cells, especially erythrocytes, leading to both normal cell-targeting toxicity and apparent antigenic silencing. Hu5F9-G4 and TTI-621 have been reported to induce adverse effects, such as anemia and thrombocytopenia (142, 143). Although it is possible to mitigate the adverse effects of Hu5F9-G4 by modifying the dosing regimen, it remains unclear whether this approach has an impact on efficacy (143). Hematological toxicity has been suggested to be Fc segment-dependent and this toxicity is due to SIRP-α-Fc fusion protein, whereas SIRP-α high-affinity monomers hardly cause any adverse effects (144–146). This suggests that optimizing the Fc structure of the anti-CD47 antibody may ameliorate these adverse effects. Regarding antigen silencing, the “don’t eat me” signal can be blocked by targeting the more restricted expression of SIRP-α. Therefore, screening antibodies or SIRP-α fusion proteins that weakly bind or do not bind to red blood cells using SIRP-α inhibitors or antibodies and the development of bispecific antibodies are crucial research avenues for future advancements in the field (**Figure 4**).

**Table 3:** Clinical trials targeting CD47- SIRP-α axis*.

Drug	Cancer types	Clinical phase	Status/outcomes	Clinical identifier	Clinical Remark
Hu5F9-G4	NHL, DLBCL, NHL, DLBCL	1	Completed	NCT03527147	To evaluate various targeted agents for relapsed/refractory aggressive NHL No results posted
	AML, MDS	1	Completed	NCT02678338	First-in-man safety and dose-finding in relapsed/refractory AML or high risk MDS Adverse events like decline in Hb and red blood cells agglutination Recipients being safely transfused
	Solid Tumor	1	Completed	NCT02216409	Well tolerated Adverse events included transient anemia, hemagglutination, hemagglutination etc.
TTI-621	HM, Solid Tumor	1	Recruiting	NCT02663518	Well-tolerated Activity as monotherapy 29% ORR for diffuse large B-cell lymphoma (DLBCL)
	Solid Tumors, Melanoma, MCC, SQC, BRCA, HPV-Related Malignant Neoplasm, Sarcoma	1	Terminated	NCT02890368	Adverse events as chills, injection site pain and fatigue Well tolerated Systemic and locoregional abscopal effects and potential as an immunotherapy
	LMS	1,2	Recruiting	NCT04996004	No results posted
TTI-622	MM	1	Recruiting	NCT05139225	Few or mild side effects No results posted
ALX148	HNC, HNSCC	2	Recruiting	NCT04675294	To estimate anti-tumor efficacy of pembrolizumab with or without ALX148 No results posted
	GAC	2,3	Not yet recruiting	NCT05002127	Design for patients progressed on or after prior HER2-directed therapy or chemotherapy No results posted
	AML	1,2	Active, not recruiting	NCT04755244	No results posted
	Higher Risk MDS	1,2	Recruiting	NCT04417517	To evaluate safety and tolerability of ALX148 combined with azacitidine No results posted
	Metastatic or Advanced Cancer, Solid Tumor, NHL	1	Active, not recruiting	NCT03013218	A first-in-human phase I trial of ALX148 Favorable hematological safety profile Adverse events as thrombocytopenia and neutropenia with ALX148 plus trastuzumab
	MSS-mCRC	2	Recruiting	NCT05167409	No results posted
	OCA	2	Not yet recruiting	NCT05467670	No results posted
AK117	Neoplasms Malignant	1	Completed	NCT04349969	To evaluate antitumor activity with relapsed or refractory advanced or metastatic solid tumors No results posted
	Neoplasms Malignant	1	Active, not recruiting	NCT04728334	To evaluate the adverse events and dose limiting toxicity in monotherapy No results posted
	AML	1,2	Recruiting	NCT04980885	To evaluate composite complete remission rate No result posted
	MDS	1,2	Recruiting	NCT04900350	No results posted
IBC0966	Advanced Malignant Tumors	1,2	Recruiting	NCT04980690	To evaluate the safety, tolerability and efficacy of IBC0966 for monotherapy
STI-6643	Solid Tumor, Relapsed or Refractory Tumor	1	Recruiting	NCT04900519	To evaluate the safety of STI-6643 for monotherapy No results posted
HX009	Advanced Solid Tumor	2	Recruiting	NCT04886271	To evaluate the ORR in 1 years by monotherapy No results posted
	Relapsed/Refractory Lymphoma	1,2	Recruiting	NCT05189093	No results posted
PF-07257876	NSCLC, SQC, CHN, OCA	1	Recruiting	NCT04881045	To evaluate the safety, pharmacokinetic, pharmacodynamic and potential clinical benefit of PF-07257876 No results posted
PF-07901801	DLBCL	2	Not yet recruiting	NCT05626322	No results posted
	OCA	2	Recruiting	NCT05261490	To improve upon the activity of pegylated liposomal doxorubicin in a safe manner No results posted
Magrolimab	FL, MZL, MCL, CLL, BCL	1	Not yet recruiting	NCT04599634	The safety and efficacy of magrolimab added to venetoclax and Obinutuzumab in relapsed and refractory indolent B-cell malignancies
	Brain Cancer	1	Recruiting	NCT05169944	No results posted
	HM	1	Recruiting	NCT03248479	To confirm the safety and tolerability of magrolimab monotherapy in a relapsed/refractory AML and MDS
	MDS, AML	1,2	Not yet recruiting	NCT05367401	No results posted
AO-176	MM	1,2	Recruiting	NCT04445701	To evaluate the safety and efficacy in Relapsed or Refractory MM No results posted
	Solid Tumor	1,2	Active, not recruiting	NCT03834948	First-in-human, dose escalation and expansion study of AO-176 No results posted
IMC-002	Solid Tumor, Lymphoma	1	Recruiting	NCT04306224	First clinical study to investigate the safety, tolerability, clinical activity of IMC-002 No results posted
	Advanced Cancer	1	Recruiting	NCT05276310	Clinical trial of IMC-002 in patients with advanced cancer failed to standard therapy No results posted
SGN-CD47M	Soft Tissue Sarcoma, CRC, HNSCC, NSCLC, BRCA, OCA, PAAD, GAC, Melanoma	1	Terminated	NCT03957096	Terminated due to sponsor decision
IBI188	Advanced Malignancies	1	Completed	NCT03763149 NCT03717103	Results submitted but not posted
CC-90002	AML, MDS	1	Terminated	NCT02641002	No sufficiently encouraging profile for further dose escalation/expansion
	HM	1	Completed	NCT02367196	No results posted
NI-1801	EOC, TNBC, NSCLC	1	Recruiting	NCT05403554	First-in-human clinical study of NI-1801 with advanced, metastatic, or recurrent solid malignancies No results posted
Gentulizumab	Solid Tumor, NHL	1	Recruiting	NCT05221385	First-in-human, escalating dose trial of gentulizumab No results posted
AUR103	Solid Tumor, AML, MDS, NHL	1	Recruiting	NCT05607199	First in human study evaluating the safety, pharmacokinetics, pharmacodynamics and efficacy of AUR103
SRF231	Solid Cancers, Hematologic Cancers	1	Completed	NCT03512340	First-in-human, dose-escalation and expansion study Results submitted but not posted

* The data source from https://www.clinicaltrials.gov and the latest update date is March 9, 2023. The meaning of abbreviations in this table are the same as in **Table 1**.

### 3.1 Specific targeting of CD47 on tumor cells

#### 3.1.1 ALX148

ALX148, also known as evorpacept, is an engineered protein consisting of a high-affinity CD47 inhibitor fused to an inactive IgG-Fc region. The inactive IgG-Fc region does not bind to the Fcγ receptor, effectively reducing hematological toxicity. In vitro experiments have elucidated ALX148 promoted macrophage phagocytosis, increased the ratio of TAMs to M1, and activated dendritic cells and T cells. In murine tumor xenograft models, ALX148 has been shown to inhibit tumor growth by enhancing the ADCP activity of various anti-tumor antibodies, such as trastuzumab. Additionally, the toxicity of ALX148 in blood cells has not been observed (147). A phase I clinical trial was designed to evaluate the efficacy of ALX148 as monotherapy or in combination with pembrolizumab or trastuzumab on solid tumors, including head and neck squamous cell carcinoma (HNSCC) and NSCLC. Patients in the single-agent ALX148 group exhibited a disease control rate of 7.7%, with a median OS of 5.2 months, whereas patients that received combination therapy exhibited a disease control rate of 16.7%, with a median OS of 20.4 months. Furthermore, ALX148 was generally well-tolerated and the hematological safety profile was favorable within the specified dose range (148). Multiple phase II clinical trials are currently ongoing, such as the NCT05002127 and NCT04675294 trials.

#### 3.1.2 AO-176

AO-176, an IgG2 antibody, is a new-generation humanized CD47 blocker that was developed to block CD47 with high specificity. AO-176 not only blocked CD47/SIRP-α interaction to induce phagocytosis of tumor cells by macrophages but also directly induced dose-dependent anti-tumor activity in murine xenograft models of lymphoma, breast cancer, ovarian cancer, and gastric cancer; furthermore, AO-176 exhibited low affinity to red blood cells and favorable tolerability in cynomolgus monkeys, rendering it a promising drug candidate (149). Two clinical trials in phase I/II, namely NCT04445701 and NCT03834948, are currently recruiting participants.

#### 3.1.3 Pep-20

Pep-20 was identified by Wang et al. as a novel peptide that specifically targets and binds to human or mouse CD47 in a dose-dependent manner, thereby triggering the effect of blocking CD47 and SIRP-α. Pep-20 has been shown to inhibit melanoma growth in subcutaneous tumor-bearing mice by significantly increasing macrophage-mediated phagocytosis and stimulating the population of intertumoral CD8^+^ T cells and the secretion of IFN-γ. Moreover, a combination of pep-20 and radiotherapy has been shown to significantly delay tumor growth or lead to complete regression of tumor. Additionally, no significant difference in red blood cell counts and hemoglobin levels have been observed between pep-20-treated mice and control mice, suggesting low hematological toxicity in mice (150). Preclinical studies of pep-20 are currently limited, and further empirical evidence is needed to substantiate these findings.

#### 3.1.4 STI-6643

STI-6643 was designed to serve as a human anti-CD47 IgG4 antibody that binds negligibly to normal cells. In vitro experiments have shown that STI-6643 did not affect the coagulation activity of human erythrocytes, even at concentrations as high as 300 μg/mL. Simultaneously, in lymphoma xenograft tumor mouse models, STI-6643 has been demonstrated to exhibit comparable anti-tumor activity to that of Hu5F9 while conserving T-cell function, thereby exhibiting promising potential for application (151).

#### 3.1.5 Bispecific antibodies

Wang et al. designed a CD47/PD-L1 bispecific antibody, namely IBI322, that selectively binds to CD47^+^ PD-L1^+^ tumor cells to trigger intense phagocytosis of tumors by macrophages and enhance T-cell activation. Notably, the affinity of IBI322 to SIRP-α is significantly lower compared to the affinity of the parental anti-CD47 antibody to SIRP-α, suggesting that IBI322 has minimal impact on CD47 single-positive cells, such as erythrocytes in cynomolgus monkeys (152). Similarly, Chen et al. designed another PD-L1/CD47 bispecific antibody, namely BisAb, which exhibited a high affinity for PD-L1, thereby enhancing tumor exposure and reducing the risk of anemia. In murine and cynomolgus monkey tumor models, BisAb has been shown to induce a unique activation of the innate immune system, involving the pattern recognition receptor-mediated type I interferon pathway, as well as antigen presentation by DCs and macrophages; this has been shown to promote the differentiation of progenitor CD8 T cells into effector T cells and alter the immune status in the TME (153). This finding has established a connection between innate and adaptive anti-tumor immunity.

This section provides an overview of the improved design of a few currently employed CD47 inhibitors which exhibit reduced hematological toxicity. These compounds have shown little hematological toxicity in preclinical studies and have demonstrated superior antitumor activity and promising applications.

### 3.2 Targeting SIRP-α in macrophages

Similar to CD47-targeting agents, anti-SIRP-α drugs inhibit CD47/SIRPα signaling in tumors; however, compared to anti-CD47 antibodies that cause antigenic silencing, they can be administered at lower doses and, owing to the limited expression of SIRP-α, are less likely to cause hematological adverse events. 1H9, a humanized anti-SIRP-α monoclonal antibody, was expressed on the cell surface of phagocytosed macrophages for an extended duration, wherein it blocked the junctional interaction between CD47 with SIRP-α. In murine models of lymphoma, the independent use of 1H9 did not induce macrophage phagocytosis; however, when combined with rituximab or cetuxima, it significantly enhanced the phagocytic and cytotoxic activity of macrophages and neutrophils against lymphoma cells. Moreover, no hematological adverse effects have been observed in cynomolgus monkeys treated with 1H9 (154). Similarly, the co-administration of KWAR23, an anti-SIRP-α antibody, and rituximab, an anti-CD20 antibody, or orsetuzumab, an anti-CD70 antibody, strongly inhibited the growth of Burkitt lymphoma in mice models (155). ADU-1805, a humanized monoclonal IgG2 antibody, binds to all known human SIRP-α alleles and effectively blocks the interaction between SIRP-α and CD47. In tumor xenograft mice, ADU-1805 triggered the repolarization of M2 macrophages to the M1 type in the TME and enhanced rituximab-induced phagocytosis of human macrophages in a concentration-dependent manner (156). This finding suggests that anti-SIRP-α antibody improved the phagocytic activity of macrophages and triggered their polarization to the M1-like phenotype. The efficacy of SIRP-α inhibitors alone may be limited; however, their combination with antibodies that target various tumor antigens could result in enhanced anti-tumor effects, thereby broadening the scope of anti-SIRP-α cancer immunotherapy.

## 4 CD39/CD73 axis

The ATP-adenosine pathway is a crucial regulator of innate and adaptive immunity in the TME. CD39 and CD73 can catalyze the hydrolysis of ATP to adenosine. CD39, also known as ectonucleoside triphosphate diphosphohydrolase 1, converts extracellular ATP and ADP into AMP. Subsequently, CD73, an ecto-5’-nucleotidase, hydrolyzes AMP to adenosine. As reported previously, high extracellular ATP levels activate infiltrating inflammatory cells and promote the formation of an anti-tumor immune environment (157), whereas production of adenosine through hydrolysis is associated with the formation of an immunosuppressive microenvironment, such as the promotion of immunosuppressive cell infiltration (158).

**Figure 5:**
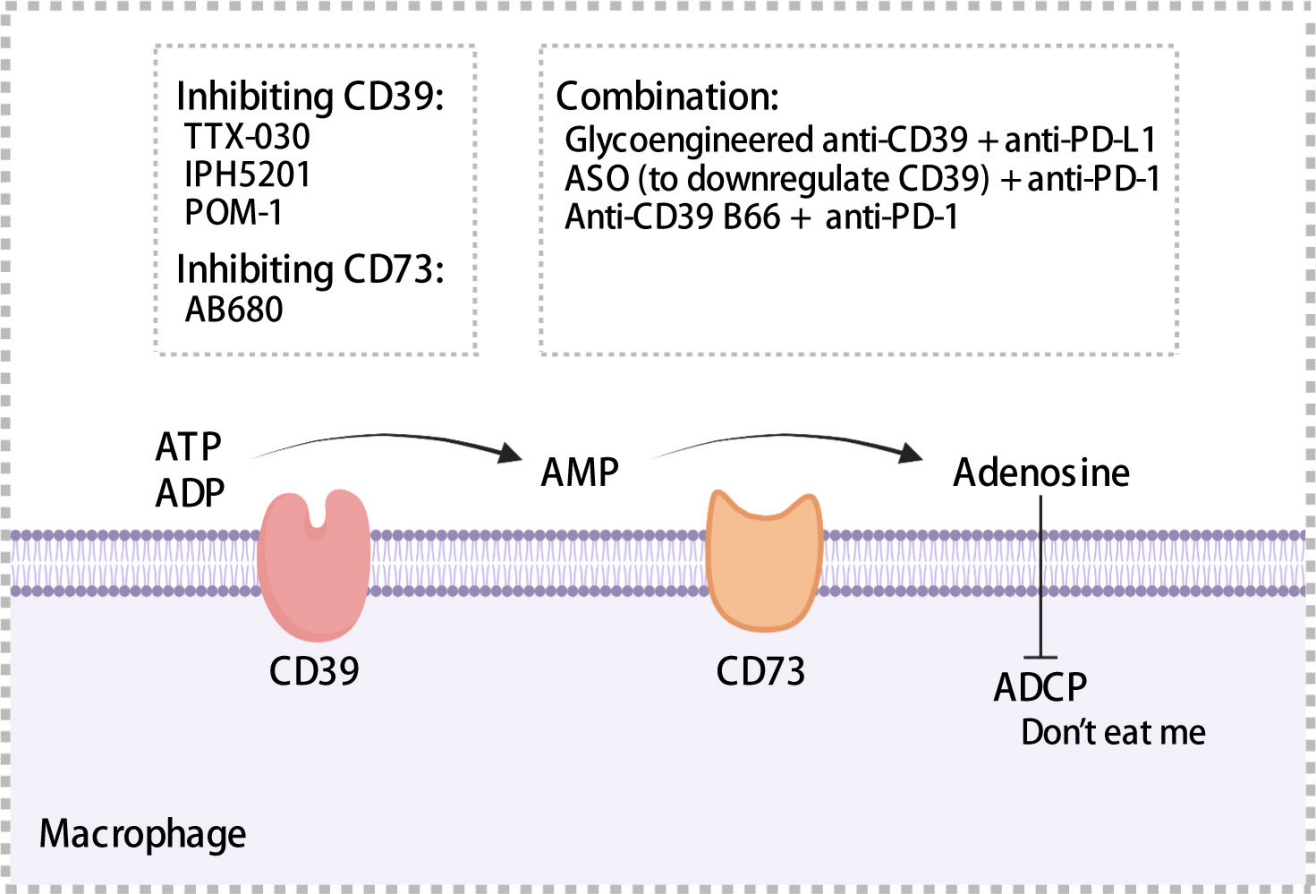
Mechanisms of CD39 and CD37. CD39 and CD73 hydrolyze extracellular ATP to adenosine, which impairs antibody-dependent cellular phagocytosis of macrophages and acts as a “don’t eat me” signal to mediate immunosuppression. A series of inhibitors targeting CD39 or CD73 have been summarized. ADCP, antibody-dependent cellular phagocytosis; ASO, antisense oligonucleotides. The figure was created with BioRender.com.

CD39 was expressed on endothelial cells, tumor cells, and immune cells such as macrophages and DCs (159–161). Moreover, CD39 was expressed in >90% of B cells and monocytes in TME (162). TAMs with high expression of CD39 that exhibit increased enzymatic activity have been observed in ovarian cancer (163). Overexpression of CD39 has been associated with shorter OS and poor prognosis using samples from HCC and chronic lymphocytic leukemia patients (164, 165). CD73 is often expressed in the bone marrow, tumors, endothelial cells, etc. In pancreatic cancer, ovarian cancer, and glioblastoma, patients with high CD73 expression exhibited a prognosis worse than that exhibited by patients with low CD73 expression (166–168). Furthermore, in pancreatic cancer, CD73 expression was significantly positively correlated with PD-L1 expression (168). In glioblastoma, a high density of CD68^+^ macrophages expressing CD73 has been observed through single cell RNA-sequencing (167). Adenosine regulated intracellular cyclic adenosine monophosphate (cAMP) levels through the A1, A2a, A2b, and A3 receptor-mediated signaling pathways, thereby affecting anti-tumor immune response (162). Adenosine binding to A2a receptors inhibited the proliferation and cytotoxic activity of effector T cells (169), impaired ADCC of macrophages, which served as a “don’t eat me” signal (**Figure 5**), and may promoted the migration of macrophages to tumors (170). Accordingly, high expression of CD39 and CD73 was associated with the formation of a tumor immunosuppressive microenvironment, which ultimately results in an impaired anti-tumor immune response.

CD39/CD73 expression on macrophages has profound impacts on anti-tumor immunity. Kynurenine produced by glioblastoma cells can activate aryl hydrocarbon receptors and upregulate the expression of CCR2 and CD39 on macrophages, thereby increasing adenosine levels and suppressing tumor-specific T cell response (171). IL-27, which is secreted by tumor-infiltrating neutrophils, has been reported to drive CD39 expression in macrophages to maintain a suppressive phenotype and increase IL-10 secretion (163). cAMP drove cAMP-response the transcription of *Cd39*, inducing its transcription by stimulating protein kinase A; this phenomenon may partially explain the mechanism underlying the upregulation of CD39 expression in TAMs (172). CircTMEM181 in HCC-derived exosomes sponged miR-488-3p in macrophages and upregulated CD39 expression on macrophages, activated the adenosine pathway, and induced M2-like polarization of the macrophages, thereby impairing anti-tumor immunity (173). Ludwig et al. demonstrated that exosomes derived from HNSCC cells were enriched in CD39, CD73 and adenosine, induced macrophages to transition to the M2 phenotype and promoted angiogenesis (174). HNSCC-derived small extracellular vesicles (sEVs) enriched in CD73 promoted CD73 expression on macrophages, triggered their polarization towards the M2 phenotype by activating the NF-κB pathway, simultaneously upregulating the expression of PD-1 and PD-L1 on macrophages. Depletion of CD73 in sEVs reversed resistance to PD-1 antibody therapy (175). These findings provide valuable insights into the regulation of CD39/CD73 on cell surfaces. Various drugs and antibodies have been developed to target CD39 and CD73. TTX-030 is a potent metamorphic inhibitor of CD39 activity and exhibited a maximum CD39 ATPase inhibition rate of 85% (176) and IPH5201 inhibited adenosine production by approximately 70%. In preclinical mouse models, the combination of IPH5201 and oxaliplatin, a chemotherapeutic agent that induced ATP production, significantly inhibited melanoma growth (177). Both drugs are currently undergoing clinical trials (NCT03884556, NCT04306900, and NCT04261075). Polyoxotungstate, an inhibitor of CD39, has been shown to increase the levels of M1 anti-tumor macrophages as well as CD8^+^ T cells and significantly inhibit the growth of CT26 colon cancer (178). AB680, a selective CD73 inhibitor, induced the proliferation and cytotoxic activity of depleted CD8^+^ T cells and significantly inhibited the growth of colon cancer established by CT26 cells; moreover, it exhibited favorable pharmacokinetic properties. Three phase I clinical trials (NCT04104672, NCT04575311, and NCT03677973) have been designed, two of which were recently concluded; however, their results are yet to be published (179). In addition, several studies have reported that CD39 or CD73 inhibitors and PD-1/PD-L1 mAbs exhibit synergistic anti-tumor effects. Zhang et al. modified an anti-CD39 IgG2 antibody by glycosylation that could deplete CD39-highly expressed immunosuppressive cells by enhancing antibody-dependent cytotoxicity; moreover, the modified anti-CD39 IgG2 and anti-PD-L1 immunotherapy exhibited favorable synergistic effects in mice models of melanoma and colorectal cancer (180). Kashyap et al. designed antisense oligonucleotides to efficiently knock down CD39 in regulatory T cells and TAMs in a mice model of breast cancer, thereby improving the response to anti-PD-1 antibody by upregulating PD-1 expression on CD8^+^ T cells (181). Li et al. reported that B66, an anti-CD39 antibody, reversed the resistance of colon cancer to PD-1 mAbs and the release of IL-18/IL-1β to inhibit colon cancer growth in mice by activating neutrophilic alkaline phosphatase 3 inflammatory vesicles, thereby elucidating another anti-tumor mechanism of anti-CD39 antibodies (182).

Targeting CD39 with therapeutic antibodies to enhance extracellular ATP signaling is a promising approach for cancer treatment and is expected to produce synergistic effects, ultimately resulting in improved tumor suppression when used in combination with chemotherapeutic agents that induce ATP release or PD-1/PD-L1 checkpoint inhibitors. In the field of antitumor immunity, CD73 or CD39 inhibitors have emerged as novel therapeutic targets that exhibit superior anti-tumor activity, improve immunosuppressive microenvironment, and demonstrate synergistic effects in combination with chemotherapy and immunotherapy in preclinical studies. These inhibitors are promising research prospects. Subsequent clinical trials are progressing to the preclinical phase, with concurrent safety assessments.

## 5 VISTA axis

VISTA, a member of the B7 family, is highly homologous to PD-1. Its ligands are primarily V-set and Ig domain-containing 3 (183) and P-selectin glycoprotein ligand-1 (184). VISTA serves as a receptor and possesses an SHP-2 binding motif, as well as multiple casein kinase 2 and phosphokinase C phosphorylation sites in the cytoplasmic structural domain. It can signal VISTA-expressing cells in a PD-1-like manner when bound to its ligand (185). Hou et al. reported that VISTA expression is higher in immune cells than in pancreatic cancer cells and endothelial cells; moreover, among immune cells in melanoma specimens from patients, the number of CD68^+^ macrophages were higher than that of CD3^+^ T and CD19^+^ B cells (186). Patients with PD-L1-positive bladder cancer exhibited higher VISTA expression than patients with PD-L1-negative bladder cancer (187, 188). Furthermore, He et al. reported a significant correlation between PD-L1 and VISTA expression, which could be synergistically employed to predict prognosis in lymphoma (189). These findings indicate that the VISTA signaling pathway has the potential as a promising target for addressing the resistance encountered in currently available immune checkpoint therapies.

## 6 Siglec-10/CD24 axis

Siglec-10, an inhibitory receptor of the immunoglobulin superfamily containing two inhibitory motifs based on the cytoplasmic immune receptor tyrosine, is widely expressed on immune cells, such as macrophages and T cells, albeit primarily on macrophages (190, 191). CD24, an adhesion molecule, is a glycoprotein with multiple N- and O-linkage sites where sialic acid can be attached *via* α-2-3- or α-2-6-links. Siglec-10 recognizes and binds to sialic acid ligands with α-2-3 or α-2-6-links, thereby triggering an inhibitory signaling cascade mediated by SHP-1 and/or SHP-2 phosphatases. This is considered a “don’t eat me” signal on tumor cells to deter macrophage phagocytosis (192, 193). The enrichment of singles-10-positive cells within tumors has been associated with poor prognosis. TAMs with high siglec-10 expression exhibit a mixed M1/M2 phenotype and immunosuppressive activity and increased levels of immunosuppressive markers such as Arg-1, IL-10, TGF-β, TIM-3, and PD-L1. Paired with the PD-1 antibody, blocking siglec-10’s binding to CD24 improved the anti-HCC activity of CD8^+^ T cells and may synergistically promote tumor apoptosis which were from fresh human specimens with HCC patients (194). Targeting siglec-10 may be a promising approach to restoring anti-tumor immunity; however, further studies are required.

## 7 Conclusion

TAMs, which are immune cells that play participate in all stages of tumors, have attracted substantial interest because of their ability to influence tumor therapy. This review outlined the findings regarding the expression and regulation of various immune checkpoints on macrophages, their impact on prognosis, and their complex interactions with immunotherapy. Moreover, we discussed how these recent insights can be translated into anti-tumor treatment strategies and potential approaches to address the poor efficacy of immunotherapy. Currently, few of the clinical trials targeting the aforementioned immune checkpoints on macrophages have shown favorable therapeutic outcomes. The majority of these trials are still in the recruitment and ongoing phases, whereas few of them have been terminated prematurely owing to inadequate efficacy, needing more investigation.

The following research directions may be useful in the future to validate clinical trial outcomes: (1) improving the transition of preclinical research to clinical research, such as through the application of humanized animal models; (2) TAM subpopulations being isolated, characterized, and correlated with clinical outcomes precisely through the application of single-cell sequencing, multicolor immunofluorescence, spatial transcriptome, and machine learning, etc. and targeting key TAM subpopulations to enhance clinical therapeutic effects, as a promising strategy; (3) reprogramming TAMs to anti-tumor macrophages using TLR and CD40 agonists or PI3K-γ inhibitors to enhance immune activity as another feasible approach; (4) incorporating M1-like phenotypic genes using CAR vectors to construct engineered macrophages for tumor treatment; the potential of CAR-M to surmount host rejection and facilitate allogeneic therapy and overcoming the challenges of tumor immune escape and enhancing the yield of CAR-M; (5) the utilization of nano-delivery systems to precisely and efficiently target TAMs; however, the safety, tolerability, and drug delivery efficiency of nanoparticles requiring further evaluation.

Further and more comprehensive studies are required to clarify the indications for implementing these strategies and their efficacy and potential adverse effects on overall anti-tumor immunity. We anticipate that enhancing the efficacy of checkpoint inhibitors through the implantation of suitable strategies that improve the blockade of immune checkpoints expressed in TAMs will be essential for improving the therapeutic efficacy of existing immunotherapies and developing novel immunotherapeutic strategies.

## Author contributions

All authors listed have made a substantial, direct, and intellectual contribution to the work and approved it for publication.
